# Free Radical Scavenging Activity of *Kielmeyera variabilis* (Clusiaceae)

**DOI:** 10.3390/molecules18022376

**Published:** 2013-02-19

**Authors:** Aline Coqueiro, Luis Octávio Regasini, Scheila Cristina Gutkoski Skrzek, Marcos Marçal Ferreira Queiroz, Dulce Helena Siqueira Silva, Vanderlan da Silva Bolzani

**Affiliations:** Department of Organic Chemistry, Institute of Chemistry, São Paulo State University, Araraquara 14800-900, Brazil; E-Mails: alinedqi@gmail.com (A.C.); regasini@gmail.com (L.O.R.); scheilagut@gmail.com (S.C.G.S.); m_marcal@hotmail.com (M.M.F.Q.); dhsilva@gmail.com (D.H.S.S.)

**Keywords:** *Kielmeyera variabilis*, Clusiaceae, antioxidant, free radical scavenging activity, DPPH^•^, ABTS^•+^, flavonoid, flavonol

## Abstract

As part of our ongoing research on antioxidant agents from Brazilian flora, we screened the free radical scavenging activity of two extracts and eight fractions of *Kielmeyera variabilis* (Clusiaceae) using DPPH^•^ (2,2-diphenyl-1-picrylhydrazyl-hydrate) and ABTS^•+^ [2,2'-azinobis(3-ethylenebenzothiazoline-6-sulfonic acid)] colorimetric assays. The ethyl acetate and *n*-butanol fractions of the leaves of *K. variabilis* displayed the strongest activity (IC_50_ of 3.5 ± 0.3 and 4.4 ± 0.2 μg mL^−1^ for DPPH^•^ and 6.6 ± 0.4 and 3.1 ± 0.1 μg mL^−1^ for ABTS^•+^, respectively). Chromatographic fractionation of the most potent fractions led to identification of three flavonols with previously described antioxidant activity, quercitrin (**1**), quercetin-3-*O*-β-glucoside (**3**), and quercetin-3-*O*-β-galactoside (**4**), and of one biflavone, podocarpusflavone A (**2**). This is the first time that the presence of these flavonoids in *Kielmeyera variabilis* has been reported.

## 1. Introduction

Clusiaceae is a tropical family of trees, shrubs, and herbs comprising approximately 50 genera and 1,200 species [1]. The chemical composition of species of this family has been extensively studied.

The genus *Kielmeyera* is found strictly in South America. Forty-seven species of this genus are known, and 45 of these species are native to the Brazilian biomes. These plants are mainly found in the Cerrado biome, located in Midwestern Brazil [2]. Many works have reported that in Brazilian folk medicine this genus is used to treat several tropical diseases, including schistosomiasis, leishmaniasis, malaria, and fungal and bacterial infections [3].

*Kielmeyera variabilis* Mart. is a tree commonly known as “malva-do-campo”, and syn. “pau-santo” in Brazil. It is distributed in the Brazilian states of Minas Gerais, São Paulo, Paraná, Rio de Janeiro, Goiás, and Piauí [4]. A previous phytochemical study on this species reported isolation of three prenylated xanthones: assiguxanthone B, 1,3,5,6-tetrahydroxy-2-prenylxanthone, and kielcorin, and also 2,5-dihydroxybenzoic acid [5].

As part of our bioprospecting program, which aims to discover antioxidant agents present in the Brazilian flora, we have screened hundreds of plants collected in the state of São Paulo and obtained promising results [6,7,8]. Among these plants, we decided to investigate the biological and chemical properties of *K. variabilis*. To our knowledge, the radical scavenging capacity of this species has not yet been reported.

Free radicals are chemically unstable species that damage lipid cells, proteins, and DNA. They result from an imbalance between the generation of reactive oxygen species (ROS) and the antioxidant protection conferred by enzymatic systems [9,10]. Free radicals cause the oxidative stress implicated in the pathogenesis of various diseases, such as cancer, diabetes, and cardiovascular diseases, and aging [9,11]. Natural products of different structural patterns can combat these free radicals [12], so screening natural product extracts is a valid strategy to discover antioxidant compounds present in microorganisms and plants [13,14,15,16].

The present study aimed to screen the extracts, fractions, and isolated compounds from the leaves and branches of *K. variabilis* for their free radical scavenging activity against the DPPH^•^ (2,2-diphenyl-1-picrylhydrazyl-hydrate) and ABTS^•+^ [2,2'-azinobis(3-ethylenebenzothiazoline-6-sufonic acid)] radicals.

In recent years, a wide range of spectrophotometric assays has been implemented to measure antioxidant capacity of foods, extracts and pure compounds, being DPPH and ABTS very useful assays to evaluate and quantify free radical scavenging activity in secondary metabolites. Most assays employ the same principle: a synthetic colored radical or redox-active compound is generated, and the ability of a biological sample to scavenge the radical or to reduce the redox-active compound is monitored by spectrophotometer, applying an appropriate standard to quantify antioxidant capacity. One approach is based on an electron transfer and involves reduction of a colored oxidant. In specification, the ABTS assay is particularly interesting in plant extracts because the wavelength absorption at 734 nm eliminates color interference, this assay is based on the generation of a blue/green ABTS^•+^ that can be reduced by antioxidants; whereas the DPPH assay is based on the reduction of the purple DPPH^•^ to 1,1-diphenyl-2-picrylhydrazine [17,18,19,20].

## 2. Results and Discussion

In the present work, we evaluated the free radical scavenging activity of the ethanol extract and four fractions resulting from the partition (hexane, ethyl acetate, *n*-butanol and aqueous methanol) of the leaves and branches of *K. variabilis* using the radicals DPPH^•^ and ABTS^•+^; which the results are summarized in Table 1.

**Table 1:** Results of the scavenging effect of *Kielmeyera variabilis* on DPPH^•^ and ABTS^•+^ radicals expressed as IC_50_ (μg mL^–1^).

Plant parts or	Extracts or	DPPH^•^	ABTS^•+^
compounds	fractions		
Leaves	ethanol	7.50 ± 0.7	7.00 ± 1.0
	hexane	>66.7	>66.7
	ethyl acetate	3.50 ± 0.3	6.60 ± 0.4
	*n*-butanol	4.40 ± 0.2	3.10 ± 0.1
	aqueous methanol	27.4 ± 2.0	27.5 ± 0.3
Branches	ethanol	13.9 ± 1.0	2.80 ± 0.2
	hexane	65.9 ± 2.5	47.2 ± 0.5
	ethyl acetate	13.4 ± 0.6	13.5 ± 1.2
	*n*-butanol	4.90 ± 0.2	3.20 ± 0.3
	aqueous methanol	46.6 ± 0.4	47.8 ± 2.0
quercitrin (1)		9.10 ± 0.5	12.2 ± 0.7
podocarpusflavone A (2)		>33.3	>33.3
quercetin-3-*O*-β-glucoside (3) +		4.10 ± 0.2	5.00 ± 0.4
quercetin-3-*O*-β-galactoside (4)			
Rutin ^a^		6.20 ± 0.3	7.40 ± 0.6

a = positive control.

Analysis of the IC_50_ values of the ethanol extracts and fractions of the leaves and branches of *K. variabilis* revealed that the most nonpolar (hexane) and the most polar (aqueous methanol) fractions displayed the lowest activity toward the two tested free radicals, whereas the ethanol extracts from the leaves and branches were more active than these fractions. The ethyl acetate and *n*-butanol fractions had similar or even better activity that of the corresponding ethanol extracts, suggesting that the potential anti-DPPH^•^ and anti-ABTS^•+^ compounds were present in the medium-polarity fractions.

For the DPPH^•^ radical, the IC_50_ value (μg mL^−1^) of the ethanol extract of the branches (13.9 ± 1.0) was comparable to that of the active ethyl acetate fraction also extracted from the branches (13.4 ± 0.6); the *n*-butanol fraction was the most active fraction obtained from the branches (4.9 ± 0.2). Among all the analyzed samples, the ethyl acetate fraction obtained from the leaves of *K. variabilis* was the most active extracted from the leaves, followed by the *n*-butanol fraction [IC_50_ (μg mL^−1^) = 3.5 ± 0.3 and 4.4 ± 0.2, respectively]. The ethanol extract of the leaves exhibited lower activity (IC_50_, μg mL^−1^ = 7.5 ± 0.7) than the medium-polarity leaves fractions, suggesting that the active substances were concentrated in the ethyl acetate and *n*-butanol fractions after partition.

For the ABTS^•+^ radical, the ethanol extract of the branches of *K. variabilis* was the most active (IC_50_, μg mL^−1^ = 2.8 ± 0.2), whilst its ethyl acetate and *n*-butanol fractions gave IC_50_ values (μg mL^−1^) of 13.5 ± 1.0 and 3.2 ± 0.3, respectively. The ethanol extract obtained from the leaves of *K. variabilis* furnished IC_50_ (μg mL^−1^) of 7.0 ± 1.0, and its ethyl acetate and *n*-butanol fractions afforded IC_50_ (μg mL^−1^) of 6.6 ± 0.4 and 3.1 ± 0.1, respectively.

The phytochemical study of the most active fractions of the leaves identified four compounds belonging to the flavonoid class. We isolated one flavonol identified as quercitrin (**1**) from the ethyl acetate fraction of the leaves, which gave IC_50_ values (μg mL^−1^) of 9.1 ± 0.5 and 12.2 ± 0.7 for DPPH^•^ and ABTS^•+^, respectively, and the biflavone podocarpusflavone A (**2**), inactive against both radicals.

**Figure 1:**
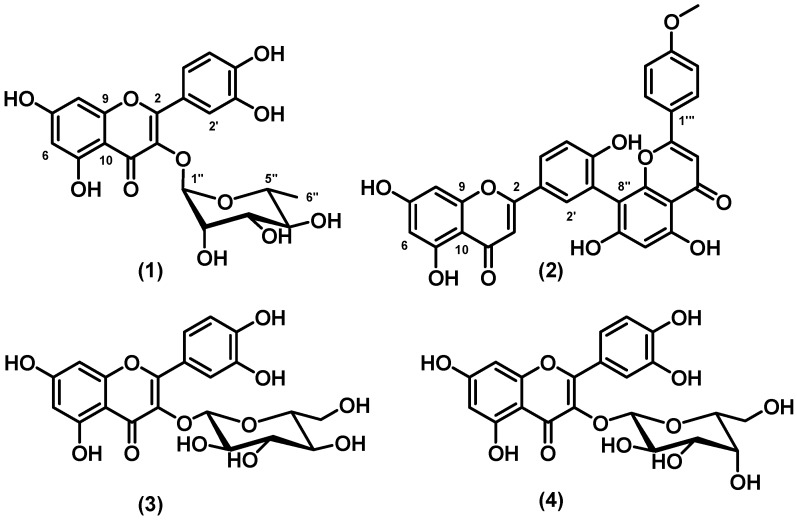
Structures of quercitrin (**1**), podocarpusflavone A (**2**), quercetin-3-*O*-β-glucoside (**3**), and quercetin-3-*O*-β-galactoside (**4**).

We isolated **1** and a mixture of two flavonols, quercetin-3-*O*-β-glucoside (**3**) and quercetin-3-*O*-β-galactoside (**4**), from the second most active fraction of the leaves of *K. variabilis* (*n*-butanol). The free radical scavenging activity of the **3** and **4** mixture was evaluated and we obtained an IC_50_ (μg mL^−1^) value of 4.1 ± 0.2 and 5.0 ± 0.4 for DPPH^•^ and ABTS^•+^, respectively. This mixture was more active than the commonly employed antioxidant rutin (IC_50_, μg mL^−1^ of 6.2 ± 0.6 and 7.4 ± 0.6 for DPPH^•^ and ABTS^•+^, respectively). Figure 1 shows the structures of flavonoids **1**–**4**.

In Nature, plants synthesize flavonoids to prevent damage by free radicals. The structures of this class of secondary metabolites contain a polyphenol structure with numerous double bonds and hydroxyl groups that can donate electrons through resonance and thus stabilize free radicals [21]. The radical scavenging properties associated with the structure of flavonoids protect against oxidative stress, reducing the risk of heart diseases, preventing cancer, and slowing down the aging process in cells responsible for degenerative diseases [22,23].

Compounds **1**, **3**, and **4** are quercetin derivatives. Quercetin is the major representative of the flavonol subclass and has received considerable attention. Quercetin and its sugar-bound, or glycosylated, forms correspond to 60–75% of flavonoids intake [24]. Many flavonoids are bound to sugars in their natural state, the *O*-glycoside form, where glycosylation can occur at any hydroxyl group, to yield a sugar. The most common glycosylated quercetin has a sugar bound at the C-3 position, as in the case of quercetin-3-*O*-glucoside. These glycosyl derivatives occur most often in Nature, and not the aglycone or the parent compound [25]. Quercetin prevents oxidation of low-density lipoproteins (LDL) by scavenging free radicals and chelating transition metal ions. As a result, quercetin can help reduce the risk of certain diseases, such as cancer, atherosclerosis, and chronic inflammation [22,25]. When the flavonol quercetin (3,5,7,3',4'-pentahydroxyflavone) reacts with a free radical, it donates a proton and becomes a radical itself, but the resulting unpaired electron is delocalized by a resonance effect, which makes quercetin a radical that has very low energy to be considered reactive [26]. Three structural groups maintain the stability of quercetin and act as antioxidant against free radicals: the B ring *o*-dihydroxyl groups, the 4-oxo group in conjugation with the 2,3-alkene, and the 3- and 5-hydroxyl groups [22]. The functional groups can donate electrons to the rings, increasing the number of available resonance forms in addition to those created by the benzene structure [26]. This agrees with our results and accounts for the activity of the isolated compounds **1**, **3**, and **4**. The predominant glucoside form is probably converted to the aglycone, which then generates one of several quercetin metabolites. However, an absorption method that can measure the level of quercetin *in vivo* has yet to be developed [27].

Analyzing the structure-radical scavenging activity relationship between the isolated compounds, the electroactivity of flavonols **1**, **3**, and **4** is due to the presence of structural features such as the *o*-dihydroxy groups (catechol group on B ring), the α,β-unsaturated carbonyl moiety, and the β-hydroxyketone groups, which enhance radical stabilization after the initial oxidation steps [28]. Compound **2**, which belongs to the flavone subclass, does not present the catechol groups in its structure, which is probably the reason why it was inactive at the tested concentrations, in agreement with literature reports [27].

## 3. Experimental

### 3.1. General

1D and 2D-NMR spectra were recorded on a Varian INOVA 500 (11.7 Tesla) spectrometer with TMS as internal standard. A Bruker Daltonics utrOTOF_Q_ with ESI operating in negative mode were used to confirm the molecular weight. For analytical analyses were used a Varian ProStar Chromatography system a with diode array detector and a Varian ProStar Chromatography unit operating in λ = 254 nm for preparative analyses. The columns were Phenomenex Luna Phenyl-Hexyl (5 μm, 250 × 4.6 mm, analytical) and a Phenomenex Phenyl-Hexyl column (10 μm, 250 × 21.20 mm, preparative). UV measures were acquired at Amersham Biosciences Ultrospec 2100 pro in λ = 517 and 734 nm using a Synergy HT-BioTeK microplate reader.

### 3.2. Plant Material

The leaves and branches of *K. variabilis* were collected in Fazenda Campininha in Mogi-Guaçú, state of São Paulo, Brazil, by Dr. Maria C.M. Young, in January 2007. It was identified by Dr. Inês Cordeiro (IBt-SMA). A voucher specimen (SP 346310) was deposited in the herbarium “Maria E.P. Kauffman” of the Botanic Institute, São Paulo, state of São Paulo, Brazil.

### 3.3. Extraction

The leaves and branches were dried, powdered and exhaustively extracted by maceration with ethanol at room temperature, in separate. After filtration, the solvent was evaporated at low temperature under reduced pressure, to yield a thick syrup. The latter was dispersed in methanol/water (4:1) and successively partitioned with hexane, ethyl acetate, and *n*-butanol. Samples of the ethanol extracts, the hexane, ethyl acetate, and *n*-butanol fractions, and the lyophilized aqueous methanol fractions were further used in the antioxidant tests.

### 3.4. Isolation and Identification of Flavonoids ***1–4***

The ethyl acetate fraction (3.5 g) was subjected to reversed-phase column chromatography (4.5 cm i.d.; 120 g of stationary phase C-18) under low pressure and eluted with a gradient system H_2_O/MeOH 95:5 to MeOH 100%, to give 17 subfractions. The subfractions were checked by TLC on silica gel F_254_ plates and grouped into nine groups of subfractions. Subfraction 1 furnished a precipitate that was filtered and washed with MeOH in an ice bath; this compound (24.5 mg) was identified as quercitrin (**1**). Subfraction 6 afforded a light yellow precipitate that was filtered and washed with MeOH and CHCl_3_, resulting in compound **2** (4.5 mg), identified as the biflavone podocarpusflavone A.

Part of the *n*-butanol fraction (3.5 g) of the leaves of *K. variabilis* was subjected to preparative gel permeation chromatography (GPC) on a Sephadex LH-20 (Pharmacia^®^) column (155 × 6.0 cm i.d.) and eluted with MeOH. Fractions (25.0 mL) were collected and checked by TLC on silica gel F_254_ plates (Merck^®^) and grouped into seven subfractions. Subfraction 2 (330 mg) was purified by reversed-phase HPLC (Phenomenex^®^ C-18 column; 5 µm) using an isocratic system MeOH/H_2_O (48:52) doped with 0.1% HOAc, flow rate of 12 mL.min^−1^ for 30 min, to yield 10.2 mg of quercitrin (**1**) and 23.7 mg of the mixture of quercetin-3-*O*-glucoside (**3**) and quercetin-3-*O*-galactoside (**4**). The molecular structures of these compounds were identified by comparison with literature data, mainly ^1^H and ^13^C-NMR δ values [29,30,31].

### 3.5. Spectroscopic Data

*Quercitrin* (**1**). ESI-MS *m/z* 447.0846 [M−H]^−^ (calcd for C_21_H_19_O_11_, 447.0927); NMR ^1^H (DMSO-*d*_6_; 500 MHz): δ 6.19 (d; 2.0 Hz; H-6); 6.38 (d; 2.0 Hz; H-8); 7.29 (d; 2.0 Hz; H-2'); 6.86 (d; 8.5 Hz; H-5'); 7.25 (dd; 2.0 and 8.5 Hz; H-6'); 5.25 (d; 1.5 Hz; H-1''); 3.97 (dd; 1.5 and 3.5 Hz; H-2''); 3.50 (dd; 3.5 and 9.3 Hz; H-3''); 3.16 (t; 9.3 Hz; H-4''); 3.20–3.23 (m; H-5''); 0.81 (d; 6.5 Hz; H-6''); 12.6 (s, 5-OH); NMR ^13^C (DMSO-*d*_6_; 125 MHz): δ 157.2 (C-2); 134.2 (C-3); 177.7 (C-4); 161.3 (C-5); 98.7 (C-6); 164.2 (C-7); 93.6 (C-8); 156.2 (C-9); 104.0 (C-10); 120.7 (C-1'); 115.6 (C-2'); 145.2 (C-3'); 148.4 (C-4'); 115.4 (C-5'); 121.1 (C-6'); 101.8 (C-1''); 70.0 (C-2''); 70.5 (C-3''); 71.2 (C-4''); 70.3 (C-5''); 17.4 (C-6'').

*Podocarpusflavone A* (**2**). ESI-MS *m/z* 551.0633 [M−H]^−^ (calcd for C_31_H_19_O_10_, 551.0978); NMR ^1^H (DMSO-*d*_6_; 500 MHz): δ 6.80 (s; H-3); 6.17 (d; 2.0 Hz; H-6); 6.43 (d; 2.0 Hz; H-8); 7.97 (d; 2.5 Hz; H-2'); 7.14 (d; 8.5 Hz; H-5'); 8.0 (dd; 2.5 and 8.5 Hz; H-6'); 6.86 (s; H-3''); 6.40 (s; H-6''); 7.66 (d; 9.0 Hz; H-2'''); 6.91 (d; 9.0 Hz; H-3'''); 6.91 (d; 9.0 Hz; H-5'''); 7.66 (d; 9.0 Hz; H-6'''); 3.73 (s; 4'''-OCH_3_); 12.95 (s; 5-OH); 13.05 (s; 5''-OH); NMR ^13^C (DMSO-*d*_6_; 125 MHz): δ 163.8 (C-2); 103.0 (C-3); 181.7 (C-4); 161.4 (C-5); 98.8 (C-6); 164.1 (C-7); 94.0 (C-8); 157.3 (C-9); 103.7 (C-10); 121.0 (C-1'); 131.3 (C-2'); 120.0 (C-3'); 159.6 (C-4'); 116.2 (C-5'); 127.8 (C-6'); 163.2 (C-2''); 103.2 (C-3''); 182.1 (C-4''); 160.5 (C-5''); 98.7 (C-6''); 161.0 (C-7''); 104.1 (C-8''); 154.5 (C-9''); 103.6 (C-10''); 123.0 (C-1'''); 128.0 (C-2'''); 114.5 (C-3'''); 162.2 (C-4'''); 114.5 (C-5'''); 128.0 (C-6'''); 55.5 (4'''-OCH_3_).

*Quercetin-3-O-β-glucoside* (**3**). ESI-MS *m/z* 463.0510 [M−H]^−^ (calcd for C_21_H_19_O_12_, 463.0877); NMR ^1^H (DMSO-*d*_6_; 500 MHz): δ 6.21 (br s; H-6); 6.41 (br s; H-8); 7.55 (d; 1.5 Hz; H-2'); 6.85 (d; 8.5 Hz; H-5'); 7.66 (dd; 1.5 and 8.5 Hz; H-6'); 5.46 (d; 7.5 Hz; H-1''); 3.24 (m; H-2''); 3.25 (m; H-3''); 3.11 (m; H-4''); 3.10 (m; H-5''); 3.57 (m; H-6''); 3.60 (m; H-6''); NMR ^13^C (DMSO-*d*_6_; 125 MHz): δ 156.4 (C-2); 133.4 (C-3); 177.5 (C-4); 161.3 (C-5); 98.8 (C-6); 164.4 (C-7); 93.6 (C-8); 156.3 (C-9); 103.9 (C-10); 122.0 (C-1'); 116.0 (C-2'); 144.9 (C-3'); 148.5 (C-4'); 115.3 (C-5'); 122.0 (C-6'); 101.0 (C-1''); 74.1 (C-2''); 76.6 (C-3''); 70.0 (C-4''); 77.5 (C-5''); 61.0 (C-6'').

*Quercetin-3-O-β-galactoside* (**4**). ESI-MS *m/z* 463.0510 [M−H]^−^ (calcd for C_21_H_19_O_12_, 463.0877); NMR ^1^H (DMSO-*d*_6_; 500 MHz): δ 6.21 (br s; H-6); 6.41 (br s; H-8); 7.59 (m; H-2'); 6.85 (d; 8.5 Hz; H-5'); 7.57 (m; H-6'); 5.37 (d; 8.0 Hz; H-1''); 3.58 (dd; 8.0 and 8.0 Hz; H-2''); 3.39 (dd; 3.0 and 8.0 Hz; H-3''); 3.67 (br s; H-4''); 3.33 (m; H-5''); 3.34 (m; H-6''); 3.35 (m; H-6''); NMR ^13^C (DMSO-*d*_6_; 125 MHz): δ 156.4 (C-2); 133.5 (C-3); 177.5 (C-4); 161.2 (C-5); 98.8 (C-6); 164.4 (C-7); 93.6 (C-8); 156.2 (C-9); 104.0 (C-10); 121.6 (C-1'); 116.3 (C-2'); 144.9 (C-3'); 148.6 (C-4'); 115.3 (C-5'); 121.6 (C-6'); 101.9 (C-1''); 71.3 (C-2''); 73.3 (C-3''); 68.0 (C-4''); 75.9 (C-5''); 60.2 (C-6'').

### 3.6. Free Radical Scavenging Activity (FRSA)

The extracts, fractions, and the flavonoids **1**–**4** were assayed in different concentrations, and their FRSA against DPPH^•^ and ABTS^•+^ was investigated.

DPPH is a stable free radical in solution and appears purple when dissolved in MeOH absorbing at 517 nm, and loss of this color indicates FRSA. The assayed samples (containing the extracts or fractions at concentrations of 66.7, 33.3, 10.0, 6.67, 3.33, 2.67, and 1.67 µg mL^−1^ or the flavonoids **1**–**4** at concentrations of 33.3, 26.7, 20.0, 13.3, 6.70, and 1.70 µg mL^−1^) were added to 0.6 μM DPPH^•^ in MeOH. The resulting mixture was incubated in the dark at 25 °C for 30 min, being the remaining DPPH^•^ determined colorimetrically at 517 nm using MeOH as blank [32,33].

The ABTS^•+^ solution was prepared by reacting 5 mL of 7 mM ABTS^•+^ aqueous solution with 88 μL of 140 mM potassium persulfate (molar ratio 1:0.35). The mixture allowed to stand in the dark at room temperature for 12–16 h before use [34]. Prior to the assay, this ABTS^•+^ stock solution was diluted with KH_2_PO_4_/K_2_HPO_4_ (100 mM, pH 7.0, diluted 1:10 before use) buffer solution (ratio 1:88) to give an absorbance of 0.414 ± 0.013 (n = 40) at 734 nm. The ABTS^•+^ solution (300 µL) was added to 96-well plates together with 10 µL of the assayed samples in the same concentrations used in DPPH^•^ test. The plates were incubated for 30 min and read at 734 nm. Lower absorbance of the reaction mixture indicated higher FRSA.

To obtain the percentage of radical redoxes (inhibition %) was used the equation: Inhibition % = [1 − (A_sample_/A) × 100], where A is the absorbance without the tested sample (only MeOH and free radical) and A_sample_ is the absorbance in the presence of the tested sample. The results are expressed as the mean IC_50_ values obtained from triplicates (IC_50_ is the concentration that provides 50% inhibition, calculated using the graph tested concentration *versus* inhibition percentage. The positive control was rutin.

## 4. Conclusions

*K. variabilis* has potential antioxidant activity against DPPH^•^ and ABTS^•+^. The ethyl acetate and *n*-butanol fractions of the leaves could be an important source of potent radical scavengers that could be used to develop novel antioxidant agents. This is the second phytochemistry study on *K. variabilis*, and it is the first time that the presence of flavonoids in this species has been reported. We were able to isolate and identify four flavonoids, quercitrin (**1**), podocarpusflavone A (**2**), quercetin-3-*O*-β-glucoside (**3**), and quercetin-3-*O*-β-galactoside (**4**) in the more electroactive fractions, which could be responsible, at least in part, for the observed free radical scavenging activity. In view of these findings, further chemical and pharmacological investigations to identify other secondary metabolites, and to evaluate the potential of this species as an antioxidant *in vivo* are recommended.
